# The Preparation of High-Purity Iron (99.987%) Employing a Process of Direct Reduction–Melting Separation–Slag Refining

**DOI:** 10.3390/ma13081839

**Published:** 2020-04-14

**Authors:** Bin Li, Guanyong Sun, Shaoying Li, Hanjie Guo, Jing Guo

**Affiliations:** 1 School of Metallurgical and Ecological Engineering, University of Science and Technology Beijing, Beijing 100083, China; libin4962337@163.com (B.L.); sunguanyong@xs.ustb.edu.cn (G.S.); 15081659279@163.com (S.L.); guojing@ustb.edu.cn (J.G.); 2 Beijing Key Laboratory of Special Melting and Preparation of High-End Metal Materials, Beijing 100083, China

**Keywords:** high-purity iron, purification, direct reduced iron, slag refining, deoxidation

## Abstract

In this study, high-purity iron with purity of 99.987 wt.% was prepared employing a process of direct reduction–melting separation–slag refining. The iron ore after pelletizing and roasting was reduced by hydrogen to obtain direct reduced iron (DRI). Carbon and sulfur were removed in this step and other impurities such as silicon, manganese, titanium and aluminum were excluded from metallic iron. Dephosphorization was implemented simultaneously during the melting separation step by making use of the ferrous oxide (FeO) contained in DRI. The problem of deoxidization for pure iron was solved, and the oxygen content of pure iron was reduced to 10 ppm by refining with a high basicity slag. Compared with electrolytic iron, the pure iron prepared by this method has tremendous advantages in cost and scale and has more outstanding quality than technically pure iron, making it possible to produce high-purity iron in a short-flow, large-scale, low-cost and environmentally friendly way.

## 1. Introduction

Pure iron, which refers to iron with very few impurities, has excellent properties such as low coercivity, high ductility, soft texture as well as good performance in thermal conductivity and electrical conductivity. High-purity iron is widely used in aerospace, radio engineering, the atomic industry and other fields. It is an important raw material for the production of precision alloys, superalloys, advanced heat-resistant alloys, amorphous alloys, soft magnetic materials, permanent magnet alloys and other materials. In recent years, pure iron has been paid more and more attention due to its extensive use and high added-value.

Pure iron is generally categorized as electrolytic iron and technically pure iron. Electrolytic iron can be prepared by electrolytic refining of high purity ferrous salt solution obtained by ion exchange or solvent extraction. The purity of a conventional electrolytic iron is about 99.9 wt.%, containing gaseous impurities such as carbon, nitrogen, oxygen, hydrogen, sulfur and chlorine of more than 500 mass ppm in total. To obtain higher-purity products, zone refining, electromagnetic levitation melting and vacuum induction melting are used to further purify electrolytic iron. Since a single purification method cannot meet the requirements of preparing ultra-high-purity iron, it is necessary to combine various purification methods. The common process is ion exchange + solvent extraction → electrolytic refining → cold-crucible melting → zone refining. Many efforts have been devoted to the preparation of high-purity iron [1]. Takaki [2] succeeded in making ultra-high-purity iron of 99.999 wt.% from Johnson Matthey pure iron by electron beam zone refining in an atmosphere of ultra-high vacuum. Abiko et al. [3,4,5] made a 7.5 kg ultra-high-purity iron ingot by careful refining of high purity electrolytic iron. The purity of the purified iron was determined to be 99.9988 wt.% by chemical analysis of 33 elements. Uchikoshi et al. [6] developed a process consisting of anion exchange in a HCl solution, hydrogen reduction and plasma arc melting for the production of semiconductor grade high-purity Fe with 99.998 wt.% purity. After improving the refining efficiency, the purity of Fe achieved 99.9993 wt.% [7].

At present, the pure iron that has been produced and applied industrially is called technically pure iron, with purity ranging from 99.6% to 99.8%. As a raw material for smelting various special alloys such as superalloys, heat-resistant alloys, precision alloys and maraging steel, technically pure iron has been widely used in metallurgical industry. Technically pure iron is produced by pyrometallurgy. Firstly, iron ore is reduced to pig iron in a blast furnace, then excessive carbon is removed by the basic oxygen furnace (BOF) or electric arc furnace (EAF), and impurities are further eliminated via a secondary refining route, through which the required purity level is achieved.

During the past few years, the demands for high-quality materials have grown more and more. Some alloys (e.g., heat-resistant alloys) use technically pure iron in smelting raw materials, but the impurities such as oxygen, phosphorus and sulfur in technically pure iron are not invariably low. These impurities cannot be eliminated readily during the alloy smelting process, leading to the situation that the alloy cannot achieve the desired performance [8]. The quality improvement of technically pure iron produced by hot metal from a blast furnace is hindered by impurity elements. Besides removing the impurities such as silicon, manganese and phosphorus, oxygen is injected to remove excess carbon in hot metal. Blowing oxygen causes an excess of oxygen to be brought into the hot metal, after which it is necessary to add aluminum or strictly control the C–O reaction using a vacuum for further deoxidation. This lengthy and complicated process, which revolves around decarbonization and deoxidation, violates the original intention of purification and makes it difficult to produce pure iron with high cleanliness. On the other hand, the concentrations of gas impurities (C+N+H+O+S) in common commercial electrolytic iron are generally high. Further refining would increase the cost and make it difficult to achieve an efficient production. The high-purity iron or ultra-high-purity iron with 99.99–99.999% purity is too expensive ($7000–200,000 US dollars/tonne) to be used on a large scale. Research and development of high-purity iron is still in the small-scale laboratory stage, and the supply cannot meet the demand. Therefore, the manufacture of pure iron has great market potential and profit margin. How to use short-process, low-cost and environmentally friendly manufacturing technology to produce high-quality pure iron is the future direction of research.

In this study, an approach of producing high-purity iron is proposed via a direct reduction of iron ore–melting separation–refining process, by which high-purity iron with purity up to 99.987% can be produced on a large scale with low cost. The process mainly includes three major steps: Step 1, the iron ore after pelletizing and roasting is reduced by hydrogen, and direct reduced iron (DRI) that is carbon-free is obtained. Some impurities such as carbon, sulfur, silicon, manganese, titanium and aluminum cannot be reduced or get into iron in this step. Step 2, the direct reduced iron is separated into gangue (slag) and metal by melting. In this step, the composition of slag is adjusted to dephosphorize, if necessary. Step 3, the high basicity slag is dosed to refining for deoxidation. Similar to the process investigated in this study, researchers [9,10,11] have studied the process of smelting pure iron with DRI in an induction furnace, which has delivered good results. However, these results still have the capacity to improve the impurities removal in pure iron, especially in solving the problem of deoxidation of pure iron, so the purity has not reached a high grade. As a comparison, the chemical compositions of pure iron in this work, typical technically pure iron and typical commercial electrolytic iron, are shown in Table 1. The purity of pure iron produced by the process has exceeded that of commercial electrolytic iron and technically pure iron. When the purity of pure iron reaches 3N level or above, it is very difficult to further improve the purity, and the increase of cost and price brought by this is an exponential growth. The pure iron obtained by this method has tremendous advantages in cost and scale compared with electrolytic iron and has more outstanding quality than technically pure iron. This is because this process takes full advantage of the purity advantage of DRI and solves the problem of deoxidization of pure iron in the context of large-scale production. This paper mainly expounds upon the experimental process and related mechanism of producing high-purity iron by this method, as well as the feasibility of industrialization.

## 2. Experimental

The raw material used in this experiment is a kind of magnetite from Shanxi, China, and the main composition is given in Table 2.

The total Fe content is about 67%, the gangue component is mainly SiO_2_ and the P and S content is relatively low. As can be seen from the composition, this iron ore is of medium grade and is characterized by homogeneous but high content of gangue, which results in more energy consumption in the melting step. In fact, lower grade iron ore is allowed to be used to reduce costs, without reducing the purity of iron.

Before being reduced by hydrogen, the magnetite powder is pelletized and then roasted in the air into oxidized pellets, in order to obtain sufficient strength and desulfurize. The oxidized pellets were prepared as follows. The magnetite powder (<0.074 mm) was thoroughly mixed with 2 wt.% binder (bentonite) and 8 wt.% water and pelletized in a disc pelletizer. The pellets were sieved so that the average diameter of the pellets was about 11 mm. The pellets were then loaded into an alumina vessel after drying for 4 h in a constant temperature drying oven. Then, the pellets were roasted in the air at a temperature of 1173 K for 30 min and then at 1493 K for 30 min for the purpose of obtaining maximum strength, according to our previous research. After testing, the mean compressive strength of pellets can reach 3000 N or above, which meets the standard of shaft furnace pellets.

**Figure 1:**
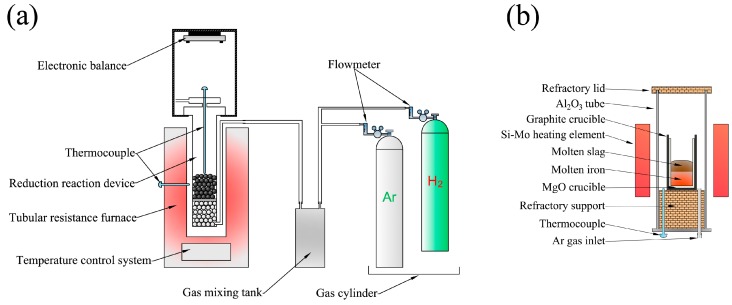
(**a**) The experimental apparatus employed for reduction of iron ore pellets and (**b**) Si-Mo resistance furnace employed to carry out the separating and refining process.

The experimental apparatus employed for reduction of pellets is shown in Figure 1a. About 500 g of oxidized pellets were weighed and placed in the reduction tube. Before charging the pellets, some alumina balls were placed at the bottom of the reduction tube for gas preheating, and a porous separator was placed between the alumina balls and the pellets. After sealing, the reduction tube was hung on the electronic balance and put into the tubular resistance furnace. The reduction tube was heated to the reaction temperature 1173 K under the protection of Ar. When the temperature became stable, the argon was turned off while the hydrogen was inserted, and the reaction began. The mass loss resulting from oxygen removal from the pellets was recorded by electronic balance over time, and the data were read once per minute for the kinetic analysis when necessary. Once the reduction finished, the reduction tube was taken out from the furnace and cooled to room temperature under the protection of argon. Ideally, almost all iron oxide can be reduced to metallic iron. Of course, the direct reduced iron (DRI) pellets with various reduction extent can be obtained by controlling the reduction time to create conditions for the melting separation and dephosphorization step.

A Si-Mo resistance furnace was employed to carry out the separating and refining process, as shown in Figure 1b. The DRI mixed with basicity slag was charged in a pure MgO crucible (99.9%). The MgO crucible with the sample was then placed inside a graphite crucible, which acted as an external holder because the MgO crucible could possibly break at high temperatures. The graphite crucible together with the MgO crucible and the sample were placed in the constant temperature zone (±1 K) of the resistance furnace to carry out the melting separation/dephosphorization experiments when the temperature increased to 1823 K. The sample was smelted at 1823 K for 30 min in a pure Ar atmosphere (99.99%) and then cooled to room temperature. The primary pure iron was obtained after removing the residual slag.

The primary pure iron together with slag were charged in another pure MgO crucible and then put into a graphite crucible. The mass of the slag was ensured to be sufficient so that the liquid slag after melting could cover the metal surface. Similarly, the graphite crucible, the MgO crucible and the sample were placed in the resistance furnace, and the secondary refining experiments were carried out when the temperature increased to 1873 K. Higher temperature can improve the fluidity of slag and promote the inclusion floatation in liquid iron. The sample was smelted at 1873 K for 30 min in the Ar atmosphere and then cooled to room temperature. The residual slag was easily peeled off from the metal, and finally the superior pure iron was obtained.

The contents of nitrogen, hydrogen and oxygen in the pure iron were determined by a N-H-O Analyzer (Manufacturer: LECO; Model: TCH600; RSD < 2%) using the pulse heating inert gas fusion–infrared absorption method, and the contents of carbon and sulfur were determined by a C-S Analyzer (Manufacturer: HORIBA; Model: EMIA-920V2; RSD < 2%) using the combustion–infrared absorption method, while other elements were determined by inductively coupled plasma–optical emission spectrometry (ICP–OES; Manufacturer: PERKINELMER; Model: OPTIMA-7000DV; RSD < 2%).

## 3. Results and Discussion

### 3.1. Removal of General Elements and the Selective Reduction of Oxides in Direct Reduction Process

**Figure 2:**
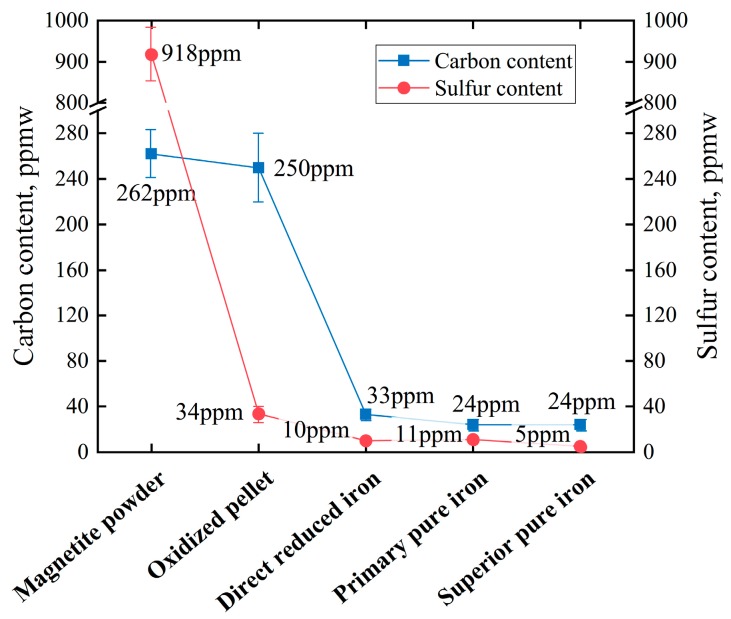
The changes in carbon and sulfur content in each step.

The reduction of oxidized pellets by hydrogen can effectively decrease sulfur and carbon in iron ore. Figure 2 shows the changes in carbon and sulfur content in each step, in which “ppmw” means parts per million by weight. As can be seen from Figure 2, the sulfur content reduces during the roasting of pellets.

The sulfur in iron ore usually exists in the form of sulfide, which can be removed by the oxidation reaction during roasting, as shown in Equations (1) and (2). The desulfurization rate of pellets during roasting can reach 96–98%, while the remaining sulfur is further reduced in the refining step.

(1) $$4{\mathrm{FeS}}_{2}+11O_{2}=2{\mathrm{Fe}}_{2}O_{3}+8{\mathrm{SO}}_{2}$$

(2) $$4\mathrm{FeS}+7O_{2}=2{\mathrm{Fe}}_{2}O_{3}+4{\mathrm{SO}}_{2}$$

(3) C+FeO=Fe+CO

The decrease of carbon content occurs mainly in the reduction step of pellets, which is the basic law of iron oxide reduction, as shown in Equation (3). In addition, the residual FeO in DRI can continue to react with carbon in the melting separation step.

The efficient and simultaneous removal of carbon and sulfur is difficult to achieve by coal-based direct reduction or gas-based reduction using a CO–H_2_ mixture. When the direct reduction iron contains a large amount of carbon, it is laborious to remove it completely in the subsequent smelting process. Although the reaction between carbon and FeO in the melting process is considered, it is almost impossible to reduce carbon to a very low level in the end. For coal-based direct reduction, the main source of sulfur is solid reducing agent, which is easy to enter the iron in reduction process, and it brings additional desulfurization burdens for subsequent refining.

**Figure 3:**
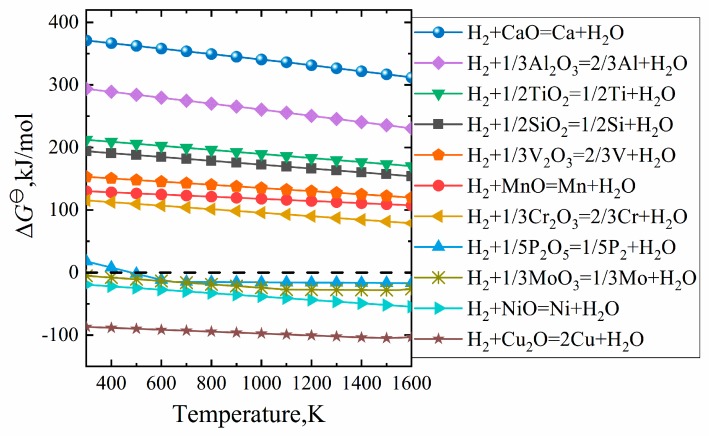
The standard Gibbs free energy change for the reactions of hydrogen with various oxides that may exist in ores.

Because of the reduction characteristics of hydrogen, the reduction process is selective, that is, the valuable element Fe can be extracted from the iron ore, while most of the valueless gangue minerals cannot be reduced into metal. The selective reduction of oxides by H_2_ can be explained by Figure 3, which shows the standard Gibbs free energy change for the reactions of hydrogen with various oxides that may exist in ores [12]. At the temperature where direct reduction occurs, i.e., 1073–1273 K, the general oxides such as manganese oxide, silicon oxide and alumina cannot be reduced into iron, while only phosphorus oxide, molybdenum oxide, nickel oxide and copper oxide can be completely reduced. The removal of phosphorus can be accomplished in subsequent steps, while the reduced copper, nickel and molybdenum are difficult to eliminate. Fortunately, the content of these oxides is relatively low in common iron ores. The blast furnace ironmaking uses coke as a reducing agent, and the temperature of blast furnace ironmaking is about 1773 K. This leads to the partial reduction of manganese oxide, silicon oxide, etc. into the molten iron, which imposes a burden on the refining process. This is why the hot metal of a blast furnace is not as pure as DRI.

### 3.2. Dephosphorization in the Melting Separation Process

**Figure 4:**
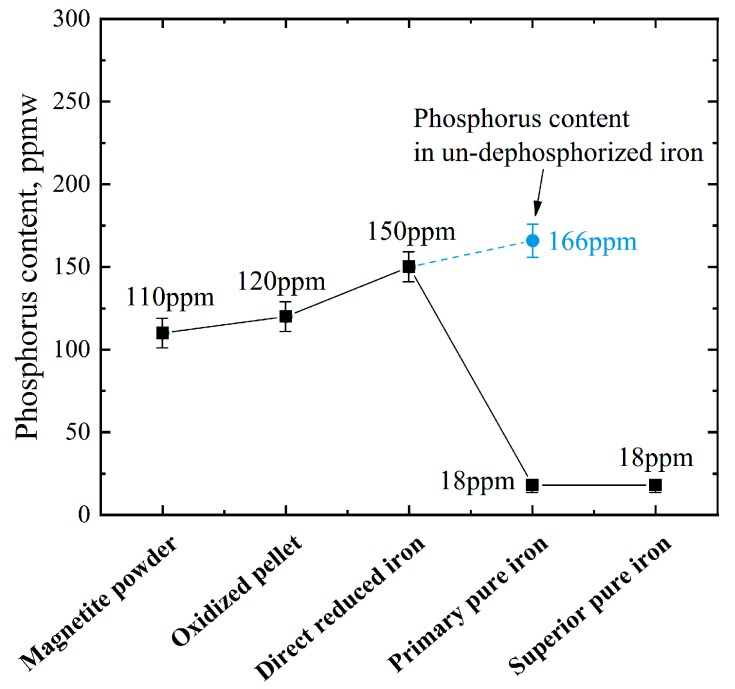
The change in phosphorus content in each step.

According to the analysis in Section 3.1, all the phosphorus in the ore is reduced after direct reduction. If the DRI is simply melted and separated at 1823 K without slagging for dephosphorization, the phosphorus content in the obtained pure iron is at 166 ppm, and if the reduced phosphorus is removed by slagging refining during the melting process, the phosphorus content drops to 18 ppm, as shown in Figure 4.

The dephosphorization reaction in the refining process is carried out between slag and metal. Previous studies by other researchers and us [13,14,15,16,17,18,19] have clearly investigated the mechanism of dephosphorization between slag and metal. Firstly, phosphorus in iron is oxidized to (P_2_O_5_) with the conditions of an oxidizing atmosphere or an oxidizing slag (FeO), as shown in Equation (4). Secondly, free CaO in high basicity slag reacts with (P_2_O_5_) to form (3CaO·P_2_O_5_), as shown in Equation (5). The formation of (3CaO·P_2_O_5_) decreases the activity of (P_2_O_5_), and this reaction is so strong that Equation (4) is promoted. Accordingly, the comprehensive dephosphorization reaction equation can be considered to be carried out by Equation (6).

(4) $$\begin{array}{l} 2[P]+5(\mathrm{FeO})=(P_{2}O_{5})+5[\mathrm{Fe}]\\ \Delta_{r}G^{⊖}=-122412+312.522T\;(J/\mathrm{mol}) \end{array}$$

(5) $$\begin{array}{l} 3({\mathrm{Ca}}^{2+}+O^{2-})+(P_{2}O_{5})=(3\mathrm{CaO}\cdot P_{2}O_{5})\\ \Delta_{r}G^{⊖}=-709890+6.150T\;(J/\mathrm{mol}) \end{array}$$

(6) $$\begin{array}{l} 2[P]+5(\mathrm{FeO})+3({\mathrm{Ca}}^{2+}+O^{2-})=(3\mathrm{CaO}\cdot P_{2}O_{5})+5[\mathrm{Fe}]\\ \Delta_{r}G^{⊖}=-832302+318.672T\;(J/\mathrm{mol}) \end{array}$$

**Figure 5:**
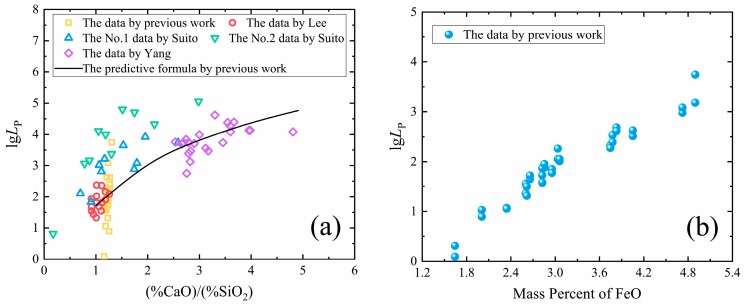
The relationship between lg*L*_P_ and slag: (**a**) the basicity (%CaO)/(%SiO_2_) and (**b**) the mass percent of FeO [19].

The phosphorus distribution ratio between slag and metal can be expressed as $L_{P}=\frac{\left({\%P}_{2}O_{5}\right)}{{\left[P\right]}^{2}}$. Basicity and FeO play an important role in dephosphorization. According to previous studies, increasing the basicity and FeO content of slag can improve the phosphorus distribution ratio, as shown in Figure 5 [19].

It is qualitatively concluded that the favorable conditions for dephosphorization are low temperature, high basicity of slag and oxidizing condition. Due to the spongy porous structure, DRI is easily oxidized during transportation and storage. Alternatively, in the actual reduction process, a certain amount of FeO can be preserved in the DRI by controlling the reduction conditions. The presence of FeO in DRI creates an innate advantage for dephosphorization, which means that no additional oxygen is needed to obtain FeO during refining. At this time, we only have to get a high basicity slag to effectively dephosphorize.

In order to quantitatively study the effect of slag composition on dephosphorization in the melting separation/dephosphorization process and further determine the suitable slag composition for dephosphorization, the thermodynamic calculation and experiments were carried out based on the designed 15 groups of slag with different CaO and FeO contents. The thermodynamic model for predicting the phosphorus distribution ratio in the melting separation process was developed based on the ion-molecule coexistence theory (IMCT) of slag [18,19]. The detailed calculation is not described repeatedly here. The composition of experimental slag used for dephosphorization is shown in Table 3.

In this experiment, the FeO in slag does not need to be additionally added, while the different FeO content contained in DRI can be controlled by changing the reducing conditions in the direct reduction step. Al_2_O_3_ is added in the slag to reduce the melting point of slag for better meltability, while MgO is added to avoid the crucible erosion. The SiO_2_ content in the DRI is relatively constant, about 9%, so other components are added proportionally according to the slag composition, which results in different total slag mass *w*_slag_ for different experimental slags. After the experiment, the phosphorus content in different pure iron samples was determined.

**Figure 6:**
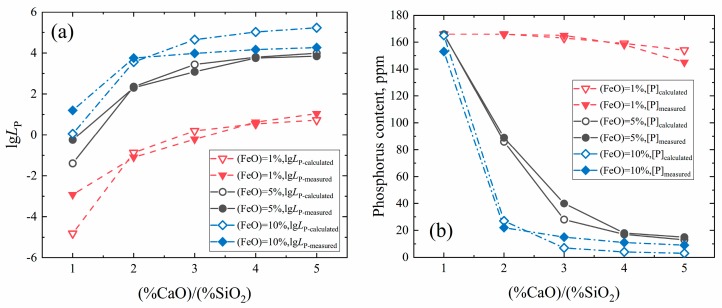
Effect of basicity (%CaO)/(%SiO_2_) and FeO content on the phosphorus distribution ratio (**a**) and phosphorus content (**b**) in pure iron.

The effect of basicity (%CaO)/(%SiO_2_) and FeO content on dephosphorization is shown in Figure 6. It can be seen from the figure that increasing the basicity can greatly promote dephosphorization, but when the basicity is more than 3, the increase of basicity has a limited effect on promoting dephosphorization. Increasing the FeO content also has a significant effect on promoting dephosphorization, but the ideal dephosphorization effect can be achieved when the FeO content reaches 5%. Excessively high FeO content is not necessary, especially when the basicity is high. Furthermore, due to the SiO_2_ content in DRI being high and relatively constant, to obtain a high basicity slag, it requires much CaO to be added, which results in a quantity of slag (see Table 3) and a lot of energy consumption in the production. The FeO content also determines the yield of iron after the dephosphorization step, and too high of an FeO content leads to an increase in iron loss.

Consequently, based on the above results and analysis, controlling the basicity at 4 and the FeO content at 5% (Slag 9) can effectively dephosphorize as well as make the yield of iron and the energy consumption within an acceptable range. In actual production, ores with low SiO_2_ should be used for production convenience and lower energy consumption. On the other hand, the necessity for the dephosphorization operation can be considered depending on the phosphorus content in ore and the purity of pure iron required.

### 3.3. Deoxidation by Secondary Refining

In metallurgy, the commonly used methods of deoxidation are precipitation deoxidation, diffusion deoxidation and vacuum deoxidation. For conventional technically pure iron, deoxidation is carried out by adding aluminum as a deoxidizer, and then the generated inclusions are removed by slagging. This results in a generally higher aluminum content in technically pure iron (see Table 1), which reduces the purity of the iron. The gas impurities such as oxygen and nitrogen in electrolytic iron are sometimes not used as an evaluation criterion. If necessary, they are usually removed by adding a small amount of carbon under the ultra-high vacuum [3]. Deoxidation for pure iron is a difficult problem because elements commonly used for deoxidation, such as carbon, silicon and aluminum, are themselves impurities for pure iron. Owing to the presence of chemical equilibrium, it is quite challenging to control the oxygen and deoxidizing elements simultaneously to a very low level.

In this experiment, the primary pure iron after melting separation/dephosphorization had an oxygen content of 300 mass ppm, and after the step of refining/deoxidizing, the amount of oxygen decreased to 10 mass ppm. Oxygen in the primary pure iron is removed by the reaction between slag and molten iron, which is to say the inclusions in iron are eliminated by the transportation into slag.

**Figure 7:**
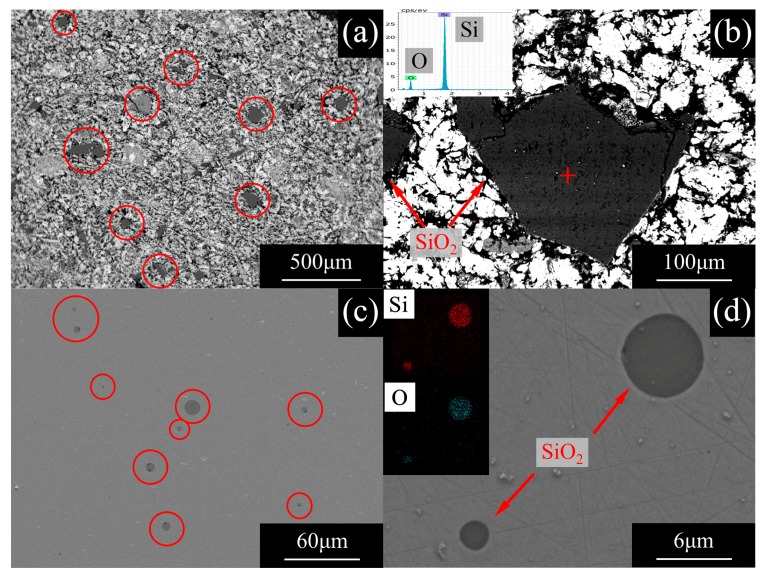
Microscopic image of SiO_2_ by SEM: (**a**,**b**) in the direct reduced iron (DRI) and (**c**,**d**) in the primary pure iron.

As the main component of the gangue in ores, SiO_2_ remains in the DRI and coexists with the reduced metallic iron particles, as shown in Figure 7a,b.

These SiO_2_ particles gather and float up to become slag during the melting process, while the iron that has a greater density becomes molten iron and separates from the slag. Research suggests that the floating velocity of particles in molten iron is proportional to the square of the radius of particles, as shown by the Stokes Equation and related correction formula [20]. For small liquid deoxidized products, taking into account the particle viscosity $\eta_{i}$, the rise velocity should be given by Equation (7).

(7) $$v=\frac{2}{3}\times \frac{(\rho_{M}-\rho_{i})gr_{i}^{2}}{\eta_{M}}\times \frac{\eta_{i}+\eta_{M}}{2\eta_{M}+3\eta_{i}}$$

In this study, the viscosity of SiO_2_ particles is much higher than that of molten iron, so the viscosity of molten iron in the formula $\frac{\eta_{i}+\eta_{M}}{2\eta_{M}+3\eta_{i}}$ can be ignored, and Equation (7) is simplified as Equation (8).

(8) $$v=\frac{2}{9}\times \frac{(\rho_{M}-\rho_{i})gr_{i}^{2}}{\eta_{M}}$$

In the above formulas, ν is the rise velocity of SiO_2_ inclusions in molten iron; $r_{i}$ is the inclusion’s radius; $\rho_{M}$ and $\rho_{i}$ are the density of the metal and of the inclusion, respectively, with values of ${7.1\;\times \;10}^{3}{\;\mathrm{kg}/m}^{3}$ and ${2.85\;\times \;10}^{3}{\;\mathrm{kg}/m}^{3}$; g is the gravity constant, with a value of ${9.8\;m/s}^{2}$; and $\eta_{M}$ is the viscosity of molten iron, with a value of 0.006 Pa·s at 1823 K.

**Figure 8:**
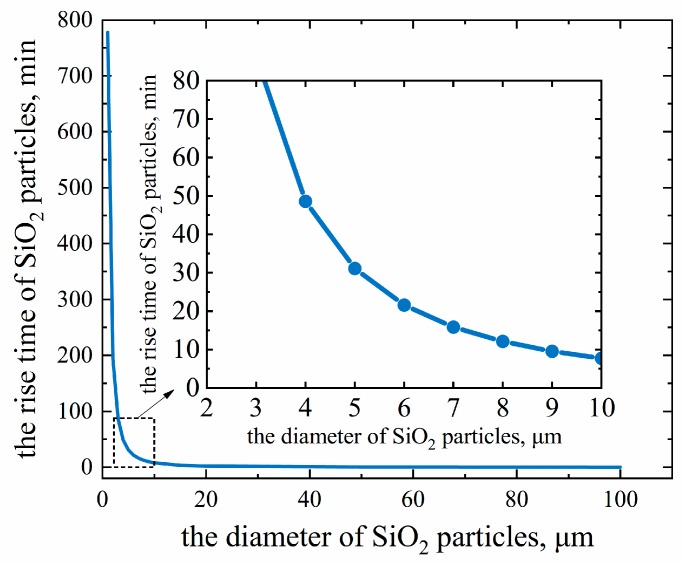
The time taken for SiO_2_ particles of different sizes in molten iron to float up.

According to the experimental conditions, if there is about 100 g of molten iron in the crucible, the depth of the molten iron is about 1.8 cm. From this, the time taken for SiO_2_ particles of different sizes in molten iron to float up can be calculated, as shown in Figure 8.

It can be seen from Figure 8 that under current experimental conditions, the duration of the melting separation process is about 20–30 min, during which not all of the SiO_2_ particles can float up into slag. Only SiO_2_ particles larger than 5 μm can rise into slag, and there are still many small-sized SiO_2_ particles left in the iron, as shown in Figure 7c,d. These SiO_2_ particles may prove to be the main source of oxygen in the primary pure iron. At this time, the task is to make suitable slag to promote the residual SiO_2_ inclusions to float and be absorbed by the slag, so as to achieve the purpose of deoxidation.

After a large number of experiments, the appropriate slag for deoxidation was determined. The composition and characteristics are shown in Table 4. The completely melting temperature and viscosity of slag were calculated using FactSage^TM^ 7.3 software. In fact, the residual SiO_2_ inclusions are equivalent to the products of the reaction of silicon and oxygen, and the driving force for their removal is highly dependent on the properties of the slag. Valdez et al. [21] and Park et al. [22] suggested that the dissolution of the inclusion into the slag is controlled by the slag phase mass transfer and that the total dissolution time of the inclusion into the slag (τ) is given by Equation (9). This result qualitatively implies that the driving force of the dissolution of the inclusion and the viscosity of the slag directly affect the removal rate of inclusion by the slag.

(9) $$\tau (s)=\frac{\rho R^{2}3\pi \alpha \eta }{2kT\Delta C},\;i.e.\;\tau (s)\propto \frac{\eta }{\Delta C}$$

where ρ is the particle density, *R* is radius of inclusion, Δ*C* is the driving force for the dissolution (concentration difference), *k* is the Boltzmann constant, *T* is the temperature, α is the ionic diameter, and η is the viscosity of the slag.

The result of the automated SEM/EDS inclusion analysis (ASPEX) is shown in Table 5, which shows that these inclusions are almost entirely SiO_2_, so a high basicity slag is necessary to provide sufficient chemical driving force. Previous studies by Ren et al. [23] and us have demonstrated the effectiveness of high basicity slag in removing SiO_2_ inclusions. According to the research on the ion-molecule coexistence theory (IMCT) for slag [24,25], the free CaO in slag has a strong binding ability toward SiO_2_, as shown in Equations (10)–(12). Consequently, an increase in the basicity of slag causes an increase in the chemical driving force for the adsorption of SiO_2_ inclusions by the slag.

(10) $$\begin{array}{l} 3({\mathrm{Ca}}^{2+}+O^{2-})+({\mathrm{SiO}}_{2})=(3\mathrm{CaO}\cdot {\mathrm{SiO}}_{2})\\ \Delta_{r}G^{⊖}=-118826-6.694T(J/\mathrm{mol}) \end{array}$$

(11) $$\begin{array}{l} 2({\mathrm{Ca}}^{2+}+O^{2-})+({\mathrm{SiO}}_{2})=(2\mathrm{CaO}\cdot {\mathrm{SiO}}_{2})\\ \Delta_{r}G^{⊖}=-102090-24.267T(J/\mathrm{mol}) \end{array}$$

(12) $$\begin{array}{l} 2({\mathrm{Ca}}^{2+}+O^{2-})+({\mathrm{Al}}_{2}O_{3})+({\mathrm{SiO}}_{2})=(2\mathrm{CaO}\cdot {\mathrm{Al}}_{2}O_{3}\cdot {\mathrm{SiO}}_{2})\\ \Delta_{r}G^{⊖}=-116315-38.911T(J/\mathrm{mol}) \end{array}$$

The excessive CaO affects the melting point and fluidity of slag, so it is necessary to add Al_2_O_3_ to lower the melting point of slag, and CaF_2_ has a great effect on reducing the viscosity of slag, which can greatly promote the adsorption of inclusions by slag, as Valdez studied. Actually, the effect of the properties such as viscosity and melting point on the adsorption capacity of slag can be considered as a physical driving force. Under the combined action of chemical driving force and physical driving force, the SiO_2_ inclusions in the primary pure iron are effectively removed.

### 3.4. Feasibility of Industrialization and Simple Estimation of Cost

**Figure 9:**
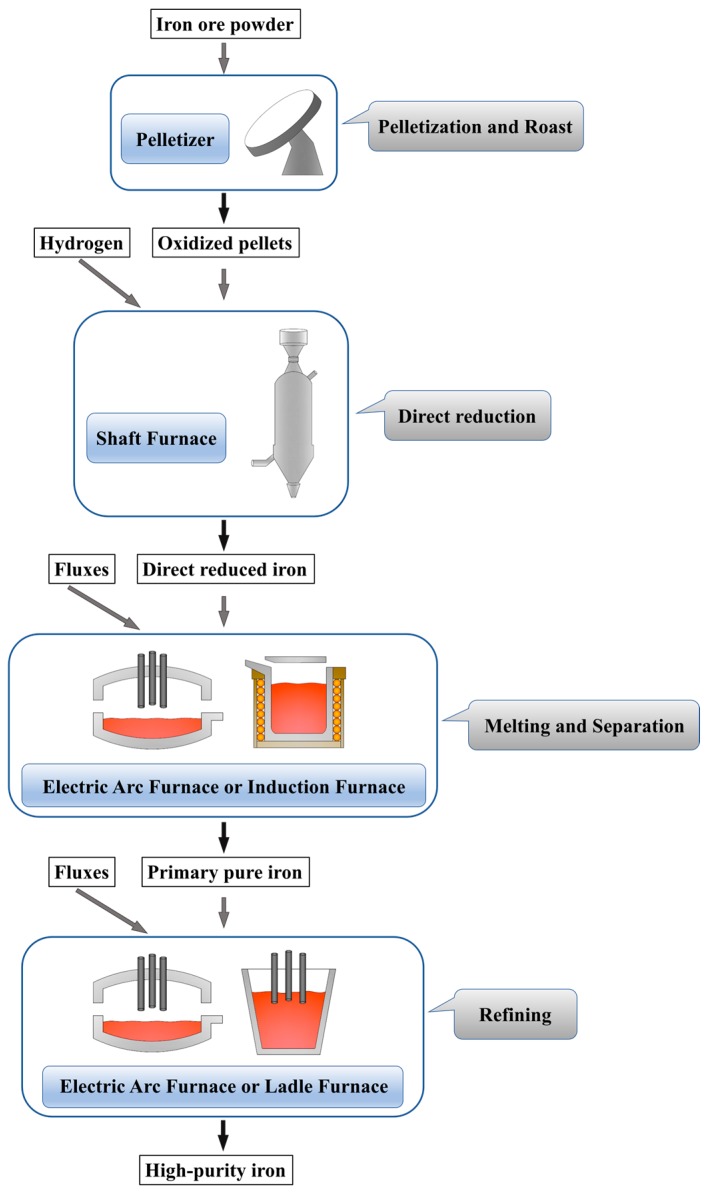
The approach proposed in this study to produce high-purity iron.

The process of producing high-purity iron is shown in Figure 9. Iron ore fines are transformed into oxidized pellets by a disc pelletizer with pelletizing and roasting functions. The direct reduction step is carried out in a shaft furnace using hydrogen as a reducing agent to produce sponge iron. Although most of the current direct reduction processes use a CO–H_2_ mixture from natural gas pyrolysis as reducing agent, hydrogen metallurgy without carbon emissions is gradually developing. Some enterprises, such as the HYBRIT project in Sweden, have carried out attempts to use pure hydrogen in metallurgy [26]. Hydrogen metallurgy is the future development direction, with the goal of reducing CO_2_ emission and developing cleaner industry. The melting process of DRI can be carried out using a mid-frequency induction furnace or an electric arc furnace, and the secondary refining can be carried out in an electric arc furnace or an LF furnace. These devices are conventional metallurgical equipment and have very mature experience in use.

The cost of high-purity iron produced by this process is shown in Table 6. It is a rough estimate, based on a project design we have done previously. In fact, the data in Table 6 are a magnified result, while the actual cost should be lower. The iron ore grade is calculated as 60% TFe. If one tonne of iron is produced, 1.667 tonnes of ore is needed. The market price of this kind of iron ore is $100 per tonne, so the ore cost is about $166.7 per tonne of iron. The price of hydrogen obtained by different processes varies greatly. Considering that more and more attention has been paid to the future use of hydrogen metallurgy, the cost of hydrogen used in industry will be lower and lower. According to the current market, the price of high-purity hydrogen with 99.999% purity is 0.7 USD/Nm^3^. It takes about 200 Nm^3^ hydrogen to produce one tonne of iron, so the total cost of hydrogen is $140 per tonne of iron. Owing to a large amount of slag with high basicity needed in the dephosphorization and melting process, about 0.6 tonnes of fluxes, such as lime and dolomite, are needed for smelting one tonne of pure iron. The price of flux is about $110 per tonne. Furthermore, 1.5 tonnes of water (recyclable), 20 Nm^3^ of natural gas (for a small amount of energy supply) and 1200 kwh of electricity are required to produce each tonne of iron. The price of energy for industrial use in China is about $0.7 per tonne of water, $0.6 per Nm^3^ of natural gas and $0.1 per kwh of electricity. Based on a plant with an annual output of 50,000 tonnes of pure iron, if there are 100 workers, and the annual salary of each worker is $20,000, then the labor cost is $40 per tonne of iron. Additionally, the annual cost of maintenance, overhaul and loss is about $1.5 million US dollars, so the average cost of maintenance is about $30 per tonne of iron.

It can be seen that the cost is at the same level as that of ordinary steel, which makes it possible to produce high-purity iron on a large scale. According to the investigation, the market price of pure iron with 99.9% purity is about $1000 US dollars/tonne, and the price of pure iron with 99.95% purity has climbed to $4000 US dollars/tonne, while the price of ultra-high-purity iron with 99.99% or higher purity reaches $7000–200,000 US dollars/tonne. Combined with the cost estimation, it can be seen that there will be huge economic benefits.

At present, the production of DRI in the world has grown rapidly, reaching 100.5 million tonnes per year in 2018. Almost all of this DRI is used as a substitute for scrap steel in the production of steel products, which is a waste of the purity of DRI. The high-purity iron produced by this approach can be used as the high-quality raw material for smelting various steels and iron-containing alloys. It only requires the addition of corresponding alloying elements to meet the requirements of products without additional purification and upgrading. Therefore, this high-purity iron and its production method will have broad application prospects for future steel manufacturing processes.

## 4. Conclusions

This study presents the research results of preparation of high-purity iron with purity of 99.987 wt.% employing a process of direct reduction–melting separation–slag refining, which can be summarized as follows.

1. The sulfur in the iron ore pellets was removed during the roasting step. Direct reduction by hydrogen produced carbon-free direct reduced iron (DRI) while avoiding the reduction of impurities such as Si, Mn, Ti, Al and V.
2. Dephosphorization was implemented simultaneously during the melting separation step by making use of the FeO contained in direct reduced iron, and the phosphorus content in primary pure iron dropped to 18 mass ppm.
3. The problem of deoxidization for pure iron was solved. Oxygen in pure iron existed as inclusions. The oxygen content of superior pure iron was reduced to 10 mass ppm by refining with a high basicity slag.
4. The cost of producing high-purity iron by this method is about $690 US dollars per tonne. Compared with electrolytic iron, the pure iron by this method has tremendous advantages in cost and scale, and has more outstanding quality than technically pure iron, which makes it possible to produce high-purity iron on a large scale.

## Figures and Tables

**Table 1:** The chemical compositions of pure iron in this work, typical technically pure iron and typical commercial electrolytic iron (wt.%).

Element	Pure Iron in This Work	Typical Technically Pure Iron	Typical Commercial Electrolytic Iron
C	0.0024	0.0030	0.0057
Si	0.0011	0.0100	0.0030
Mn	0.0003	0.0400	0.0003
P	0.0018	0.0021	0.0005
S	0.0005	0.0016	0.0018
Cr	0.0009	0.0100	0.0003
Ni	0.0005	0.0100	0.0032
Al	0.0015	0.0150	0.0011
Ti	0.0003	0.0011	0.0009
Cu	0.0001	0.0100	0.0015
Mo	0.0007	0.0020	0.0004
V	0.0005	0.0020	0.0003
N	0.0015	0.0025	0.0038
H	0.0001	0.0001	0.0013
O	0.0010	0.0030	0.0574
Purity of Fe	99.9868	99.8876	99.9185

**Table 2:** The main composition of magnetite (wt.%).

TFe	FeO	Fe_2_O_3_	SiO_2_	CaO	MnO	V_2_O_5_	P_2_O_5_	S
67.37	30.48	62.37	6.62	0.26	0.11	0.04	0.03	0.09

“TFe” indicates the total mass concentration of Fe.

**Table 3:** The composition of slag used for dephosphorization.

No.	CaO/SiO_2_	FeO	CaO	SiO_2_	Al_2_O_3_	MgO	*w*_slag_
Slag 1	1	1%	34.5%	34.5%	25%	5%	26.09 g
Slag 2	2	1%	46%	23%	25%	5%	39.13 g
Slag 3	3	1%	51.75%	17.25%	25%	5%	52.17 g
Slag 4	4	1%	55.2%	13.8%	25%	5%	65.22 g
Slag 5	5	1%	57.5%	11.5%	25%	5%	78.26 g
Slag 6	1	5%	32.5%	32.5%	25%	5%	27.69 g
Slag 7	2	5%	43.33%	21.67%	25%	5%	41.53 g
Slag 8	3	5%	48.75%	16.25%	25%	5%	55.38 g
Slag 9	4	5%	52%	13%	25%	5%	69.23 g
Slag 10	5	5%	54.17%	10.83%	25%	5%	83.1 g
Slag 11	1	10%	30%	30%	25%	5%	30 g
Slag 12	2	10%	40%	20%	25%	5%	45 g
Slag 13	3	10%	45%	15%	25%	5%	60 g
Slag 14	4	10%	48%	12%	25%	5%	75 g
Slag 15	5	10%	50%	10%	25%	5%	90 g

**Table 4:** The composition and characteristic of slag used for deoxidation.

CaO	SiO_2_	Al_2_O_3_	CaF_2_	MgO	CaO/SiO_2_	Completely Melting Temperature	Viscosity
46.2%	6.6%	23.1%	20%	4.1%	7	1736.1 K	0.0404 Pa·s

**Table 5:** The characteristic statistics of inclusions in the primary pure iron.

Average Diameter	Amount	Average Composition	
3.05 μm	56.06 /mm^2^	Si	33.98 atom %
		O	66.02 atom %

**Table 6:** The estimated cost of pure iron produced by this process (US dollar/tonne iron).

Raw Material Cost			Energy Cost			Running Cost		Sum
Ore	Reductant	Flux	Power	Gas	Water	Labor	Maintenance	690
170	210	70	120	20	20	50	30	
